# Supplementary material for: Use of Next Generation Sequencing to study two cowpox virus outbreaks
Source: PeerJ. 2019 Mar 1;7:e6561. doi: 10.7717/peerj.6561 (PMC6398431; doi:10.7717/peerj.6561)
Supplement: Table S1 [file peerj-07-6561-s001.doc]

| Orthopoxvirus  species | Virus name(indented – same HA sequence) | Enumeration | Year isolated | Geographic origin | Host | Genbank  Accession HA | Genbank  Accession genome NGS | ORF  bases |
| --- | --- | --- | --- | --- | --- | --- | --- | --- |
| Ectromelia | ECTV-Mos | E001 |  | Russia: Moscow | mouse |  | AF012825 | 846 |
|  | ECTV-mhl |  | 1959 | United Kingdom: Mill Hill | mouse | AF375091 |  | 846 |
|  | ECTV-US#33221 |  | 1995 | USA | mouse | AY902302 |  | 846 |
|  | ECTV-MPV-CC |  |  |  | mouse | DQ003024 |  | 846 |
|  | ECTV-ERPV |  | 1987 | China | human |  | JQ410350 | 846 |
|  | ECTV-K1 (Ect2) | E002 | 1989x |  | mouse | Z99054 |  | 840 |
|  | ECTV-MP-2 | E003 | 1983 | Germany: Munich | mouse | AY902304 |  | 846 |
|  | ECTV-silberfuchs |  | 1990 | Czech Republic | silver fox | AY902306 |  | 846 |
|  | ECTV-Naval | E004 | 1996 | USA | mouse |  | KJ563295 | 846 |
|  | ECTV-US#4619 |  | 1995 | USA | mouse | AY902303 |  | 846 |
|  | ECTV-K1 (Ect1) | E005 | 1989x |  | mouse | Z99053 |  | 846 |
| Monkeypox  Central Africa | MPXV-74-226 | M001 | 1974 | Democratic Republic of the Congo | human | AF375099 |  | 942 |
|  | MPXV-congo8 |  | 1970 | Democratic Republic of the Congo | human | AF375104 | KJ642613 | 942 |
|  | MPXV-RCG2003-358 |  | 2003 | Republic of Congo | human |  | DQ011154 | 942 |
|  | MPXV-Zaire 1979-005 |  | 1979 | Zaire | human |  | DQ011155 | 942 |
|  | MPXV-790005 |  | 1979 | Democratic Republic of the Congo | human | AF375100 |  | 942 |
|  | MPXV-03-cng |  | 2003 | Democratic Republic of the Congo | ?? | AY944032 |  | 942 |
|  | MPXV isolates (n=21) |  | 2001-2004 | Democratic Republic of the Congo | human | DQ443505 to  DQ443525 |  | 942 |
|  | MPXV isolates (n=5) |  |  | Democratic Republic of the Congo | human |  | JX878407 JX878423-25 JX878429 | 942 |
|  | MPXV Yandongi |  | 1985 | Democratic Republic of the Congo | human |  | KC257460 | 942 |
|  | MPXV Sudan |  | 2005 | Democratic Republic of the Congo | human |  | KC257459 | 942 |
|  | MPXV Boende |  |  | Democratic Republic of the Congo | human |  | KP849469 | 942 |
|  | MPXV-cncr | M002 | 1981x | Democratic Republic of the Congo | human | AF375102 |  | 942 |
|  |  |  |  |  |  |  |  |  |
|  | MPXV isolates (n=29) | M003 | 2001-2004 | Democratic Republic of the Congo | human | DQ443476 to  DQ443504 |  | 942 |
|  | MPXV isolates (n=17) |  |  | Democratic Republic of the Congo | human |  | JX878408-416  JX878418-422  JX878426-428 | 942 |
|  | MPXV Yambuku |  | 1985 | Democratic Republic of the Congo | human |  | KP849471 | 942 |
|  | MPXV Gabon |  | 1988 | Gabon | human |  | KJ642619 | 942 |
|  | MPXV Cameroon |  |  | Cameroon | human |  | KJ642618 | 942 |
|  | MPXV Ikubi |  |  | Democratic Republic of the Congo | human |  | KJ642612 | 942 |
|  | MPXV mpv |  | 1997 | Democratic Republic of the Congo | human | AF375096 |  | 942 |
|  | MPXV isolate DRC 07-0104 | M004 | 2006 | Democratic Republic of the Congo | human |  | JX878417 | 942 |
|  | MPXV Zaire 77-0666 | M005 | 1977 | Democratic Republic of the Congo | human | Z99052 |  | 942 |
|  | MPXV-squir | M006 | 1980 | Democratic Republic of the Congo | squirrel | AF375112 |  | 942 |
|  |  |  |  |  |  |  |  |  |
| Monkeypox  West Africa | MPXV-3945 | M007 | 1978 | Benin | human | AF375098 |  | 942 |
|  | MPXV W-Nigeria |  | 1978 | Nigeria |  |  | KJ642615 |  |
|  | MPXV-utc | M008 | 1965 | The Netherlands: Utrecht | monkey | AF375113 | KJ642614 | 942 |
|  | MPXV-wmp |  | 1961 | USA: Washington, DC | monkey | AF375114 |  | 942 |
|  | MPXV-pvm |  | 1958 | Denmark: Copenhagen | monkey | AF375111 |  | 942 |
|  | MPXV-pch |  | 1968 | France: Paris | chimpanzee | AF375110 | KJ642616 | 942 |
|  | MPXV_US2003_039 |  | 2003 | USA | human | DQ011157 |  | 942 |
|  | MPXV_US2003_044 |  | 2003 | USA | prairie dog | DQ011153 |  | 942 |
|  | MPXV61_walter reed AIR7-61 |  | 1961 | USA |  | AY944031 | AY603973 | 942 |
|  | MPXV03 |  | 2003 | USA | human | AY944030 |  | 942 |
|  | MPXV-mcc |  | 1961 | USA: Washington, DC | monkey | AF375109 |  | 942 |
|  | MPXV-cpn |  | 1958 | Denmark: Copenhagen | monkey | AF375108 |  | 942 |
|  | MPXV-cpcwn1 |  | 1958 | Denmark: Copenhagen | monkey | AF375107 |  | 942 |
|  | MPXV-cpcr |  | 1958 | Denmark: Copenhagen | monkey | AF375106 |  | 942 |
|  | MPXV-cope |  | 1958 | Denmark: Copenhagen | monkey | AF375105 | AY753185 | 942 |
|  | MPXV-266 |  | 1970 | Sierra Leone | human | AF375097 | AY741551 | 942 |
|  | MPXV-Cote dIvoire |  | 1971 | Ivory coast | human |  | KP849470 | 942 |
|  | MPXV-1970-184 |  | 1970 | Liberia | human |  | DQ011156 | 942 |
|  | MPXV-0187 |  | 1970 | Liberia | human | AF375094 |  | 942 |
|  | MPXV-Cote dIvoire |  | 2012 | Ivory coast | human |  | KJ136820 | 942 |
|  | MPXV-082 | M009 | 1971 | Nigeria | human | AF375095 | KJ642617 | 942 |
|  | MPXV-cncw-n1 | M010 |  | Congo-8 | human | AF375103 |  | 942 |
| Vaccinia | VACV_Tian Tan | V001 |  |  |  |  | AF095689 | 948 |
|  | VACV_IHDJ | V002 |  |  |  | M14783 |  | 948 |
|  | VACV_Cantagalo | V004 | 1999 | Brazil | cow | AF229247 | KT013210 | 927 |
|  | VACV_Aracatuba | V005 | 2002x | Brazil |  | AY523994 |  | 927 |
|  | VACV_LC16m8 | V006 |  |  |  |  | AY678275 | 933 |
|  | VACV_LC16mO |  |  |  |  |  | AY678277 | 933 |
|  | VACV_lst | V007 |  | United Kingdom |  | AF375124 |  | 933 |
|  | VACV-IOC | V008 |  | Brazil |  | AF229248 |  | 927 |
|  | VACV_MVA Acambis clone 3000 | V009 |  |  |  |  | AY603355 | 948 |
|  | VACV_MVA I721 |  |  |  |  |  | DQ983236 | 948 |
|  | VACV_MVA BN |  |  |  |  |  | DQ983238 | 948 |
|  | VACV_Strain Ankara |  |  |  |  |  | U94848 | 948 |
|  | VACV_MVA Acambis clone 2000 | V010 |  |  |  |  | AY313847 | 930 |
|  | VACV_MVA Acambis clone 3 | V011 |  |  |  |  | AY313848 | 930 |
|  | VACV_Dryvax clone 9 | V012 |  |  |  |  | JN654976 | 930 |
|  | VACV_len | V013 | 1975 |  |  | AF375123 |  | 933 |
|  | VACV-Cop | V014 |  | Copenhagen |  |  | M35027 | 948 |
|  | VACV-3737 | V015 |  |  |  |  | DQ377945 | 933 |
|  | RPXV-rpv-rev | V016 |  | Netherlands: Utrecht | rabbit | AF375118 |  | 939 |
|  | RPXV-u23 | V017 | 1940x | The Netherlands: Utrecht | rabbit | AF375119 |  | 939 |
|  | BPXV-81 | V018 | 1985 | India | buffalo | AF375078 |  | 945 |
|  | BPXV-3906 | V019 | 1985 | India | buffalo | AF375077 |  | 945 |
|  | VACV-COP-2 | V020 |  |  |  | Z99066 |  | 930 |
|  | VACV-Malbran | V021 | 1945 |  |  | AY146624 |  | 930 |
|  | VACV-VVHA02 | V022 |  |  |  | AY902305 |  | 948 |
|  | VACV-koppe | V023 |  | Denmark |  | AF375122 |  | 945 |
|  | VACV-LS1 | V024 |  | United Kingdom |  | AY944027 |  | 933 |
|  | VACV-LIVP-2 | V025 |  |  |  | Z99045 |  | 948 |
|  | VACV-LIVP-1 | V026 |  |  |  | Z99046 |  | 948 |
|  | VACV-WR | V027 |  |  |  |  | AY243312 | 945 |
|  | VACV-lis107 | V028 |  | United Kingdom |  |  | DQ121394 | 942 |
|  | VACV-lis Butantan | V029 | 2009 | Brasil |  |  | KX061501 | 933 |
|  | VACV-LOR2602 | V030 |  | Brazil | human | DQ810281 |  | 927 |
|  | VACV-RIA2548 |  |  | Brazil | human | DQ848593 |  | 927 |
|  | VACV-RIA2549 |  |  | Brazil | human | DQ810280 |  | 927 |
|  | VACV-RIA2550 |  |  | Brazil | human | DQ848592 |  | 927 |
|  | VACV-LAG2322 |  |  | Brazil | human | DQ848589 |  | 927 |
|  | VACV-LAG2324 |  |  | Brazil | human | DQ848590 |  | 927 |
|  | VACV-LAG2288 |  |  | Brazil | human | DQ810275 |  | 927 |
|  | VACV-GUA2432 |  |  | Brazil | human | DQ810279 |  | 927 |
|  | VACV-CHP2342 |  |  | Brazil | human | DQ810278 |  | 927 |
|  | VACV-SIL2311 |  |  | Brazil | human | DQ810277 |  | 927 |
|  | VACV-SIL2382 |  |  | Brazil | human | DQ848591 |  | 927 |
|  | VACV-PIQ2303 |  |  | Brazil | human | DQ810276 |  | 927 |
|  | VACV-Cantagalo MU-7 |  |  | Brazil |  | FJ545689 |  | 927 |
|  | VACV-Serro 2 |  | 2005 | Brazil | human |  | KF179385 | 927 |
|  | VACV-DUKE | V031 | 1970 | USA | human |  | DQ439815 | 930 |
|  | VACV-CVA-BN | V032 |  | Turkey |  |  | AM501482 | 948 |
|  | VACV-WAU86/88-1 | V033 |  |  |  |  | KF866253 | 933 |
|  | HSPV-MNR-76 | V034 | 1976 | Mongolia | horse |  | DQ792504 | 945 |
|  | VACV Colombia (n=3) | V035 | 2014 | Colombia | human | KU950327 |  | 945 |
|  | VACV Tashkent TKT3 | V036 |  |  |  |  | KM044309 | 970 |
|  | VACV Tashkent TKT4 |  |  |  |  |  | KM044310 | 970 |
|  | VACV Mulford | V037 | 1902 | USA | horse |  | MF477237 | 939 |
| Cowpox | CPXV_AUS1999-867 | C001 | 1999 | Austria | cat | AF377884 | HQ407377 | 957 |
|  | CPXV_FIN/T2000 | C002 | 2000 | Finland | human | AY366477 | HQ420893 | 948 |
|  | CPXV_FIN/E1989 |  | 1989 | Finland | human | AY366478 |  | 948 |
|  | CPXV_FIN/K2009 |  | 2009 | Finland | human | KR054112 |  | 948 |
|  | CPXV_GRI | C003 | 1990 | Russia: Moscow | human | Z99047 | X94355 | 945 |
|  | CPXV-89-1 | C004 | 1989 | Germany: Mannheim | cat | AF375084 |  | 954 |
|  | CPXV-89-5 | C005 | 1989 | Germany: Ulm | cat | AF375086 |  | 954 |
|  | CPXV-90-1 | C006 | 1990 | Germany: Deisenhofen | cat | AF375087 |  | 951 |
|  | CPXV 91/1 | C007 | 1991 | Germany: Landsberg | cat | AY902253 |  | 951 |
|  | CPXV_GER1991_3 |  | 1991 | Germany: Munich | human | AY944028 | DQ437593 | 951 |
|  | CPXV-FM2292 |  | 2011 | Germany: Baden-Württemberg | Microtus arvalis |  | LN864566 | 951 |
|  | CPXV-Tuebingen | C008 | 1979 | Germany: Tuebingen | elephant | AY902252 |  | 963 |
|  | CPXV_EP-3 | C009 | 1977 | Germany: Frankfurt | elephant | AY902277 |  | 960 |
|  | CPXV-66/01 |  | 2001 | Germany: Goettingen | human | AY902278 |  | 960 |
|  | CPXV-EP-Giessen |  |  | Germany: Giessen | elephant | AY902279 |  | 960 |
|  | CPXV Ger/2010/Cat |  | 2010 | Germany: Nordhausen | cat |  | LT896729 | 960 |
|  | CPXV Ger/2014/Cat2 |  | 2014 | Germany: Nordhausen | cat |  | LT896725 | 960 |
|  | CPXV 98/2 | C010 | 1998 | Germany: Eckental | human | AY902269 | HQ420897 | 960 |
|  | CPXV-89-4 | C011 | 1989 | Germany: Neuenrade | cat | AF375085 |  | 960 |
|  | CPXV-EP-2 | C012 | 1975 | Germany: Ansbach | elephant | AF375090 |  | 948 |
|  | CPXV-EP Wien | C013 | 1974 | Austria: Wien | elephant | AF377886 |  | 945 |
|  | CPXV-1266-98 | C014 | 1998 | Austria | human | AF377878 |  | 945 |
|  | CPXV-641-94b |  | 1994 | Austria | cat | AF377882 |  | 945 |
|  | CPXV-610-97b |  | 1997 | Austria | cat | AF377881 |  | 945 |
|  | CPXV-531-92b |  | 1992 | Austria | cat | AF377880 |  | 945 |
|  | CPXV-524-97b |  | 1997 | Austria | cat | AF377879 |  | 945 |
|  | CPXV_667_94b |  | 1994 | Austria | cat | AF377883 |  | 945 |
|  | CPXV HumGra07/1 |  | 2007 | Austria: Graz | human |  | KC813510 | 945 |
|  | CPXV-1218-00 | C015 | 2000 | Austria | human | AF377877 |  | 939 |
|  | CPXV- cowHA13 | C016 | 1997 | Germany: Berlin | beaver | AY902260 |  | 936 |
|  | CPXV-KR58 | C017 | 1958 | Switzerland | bovine | AF375083 |  | 942 |
|  | CPXV-Ratte Moskau |  | 1977 | Russia: Moscow | rat | AY902263 |  | 942 |
|  | CPXV-EP-1 |  | 1971 | Germany: Augsburg | elephant | AY902264 | KY463519 | 942 |
|  | CPXV-EP 3297 |  | 1973 | The Netherlands | elephant | AY902265 |  | 942 |
|  | CPXV-P2 Vero |  |  | The Netherlands | human | AY902266 |  | 942 |
|  | CPXV-EP-3299 |  | 1973 | The Netherlands | elephant | AY902267 |  | 942 |
|  | CPXV-EP-5 | C018 | 1988 | Germany: Erfurt | elephant | AY902283 |  | 942 |
|  | CPXV-EP-8 |  | 2000 | Germany: Bad Langensalza | elephant | AY902284 |  | 942 |
|  | CPXV-81/01 |  | 2001 | Germany: Munich | cat | AY902285 |  | 942 |
|  | CPXV-EP-7 |  | 2000 | Germany: Bad Langensalza | elephant | AY902307 |  | 942 |
|  | CPXV Ger/2012/Alpaca |  | 2012 | Germany: Rositz | alpaca |  | LT896726 | 942 |
|  | CPXV Ger/2015/Human1 (TAH) |  | 2015 | Germany: Leipzig | human |  | LT896720 | 942 |
|  | CPXV-EP-Riems | C019 | 1980 | Germany: Berlin | elephant | AY902286 |  | 936 |
|  | CPXV-OPV 85 Hamburg | C020 | 1985 | Germany: Hamburg | cat | AY902298 |  | 924 |
|  | CPXV-Nancy | C021 | 2001 | France: Nancy | human | AY902287 | HQ420894 | 918 |
|  | CPXV-Schwede II 2058-90 | C022 | 1990 | Sweden: Lund | human | AY902297 |  | 903 |
|  | CPXV-Schwede I 1971-90 | C023 | 1990 | Sweden: Malmoe | human | AY902296 |  | 900 |
|  | CPXV-Schwede III | C024 | 2001 | Sweden | human | AY902288 |  | 903 |
|  | CPXV_GER1990_2 | C025 | 1990 | Germany: Bonn | human |  | HQ420896 | 903 |
|  | CPXV-90-5 | C026 | 1990 | Germany: Honigsee | cat | AF375088 |  | 903 |
|  | CPXV 90/3 |  | 1990 | Germany: Groemitz | cat | AY902258 |  | 903 |
|  | CPXV 90/4 |  | 1990 | Germany: Groemitz | dog | AY902259 |  | 903 |
|  | CPXV-98/4 | C027 | 1998 | Germany: Goettingen | human | AY902271 |  | **816** |
|  | CPXV-EP-2501 | C028 | 1972 | The Netherlands | elephant | AY902300 |  | 921 |
|  | CPXV Shiva | C029 | 2009 | Germany: Muenchen | human | FJ654467 |  | 924 |
|  | CPXV Ratpox09 |  | 2009 | Germany: Marl | rat |  | LN864565 | 924 |
|  | CPXV Boa |  | 2009 | Germany: Krefeld | boa |  | This study | 924 |
|  | CPXV DG |  | 2009 | France: Compiegne | human | FJ754357 |  | 924 |
|  | CPXV LP |  | 2009 | France: Compiegne | human | FJ754356 |  | 924 |
|  | CPXV KP |  | 2009 | France: Compiegne | human | FJ754355 |  | 924 |
|  | CPXV HumKre08/1 |  | 2008 | Germany: Krefeld | human | GQ260461 | KC813512 | 924 |
|  | CPXV HumAac09/1 |  | 2009 | Germany: Aachen | human | JF330118 | KC813508 | 924 |
|  | CPXV RatAac09/1 |  | 2009 | Germany: Aachen | rat |  | KC813501 | 924 |
|  | CPXV RatKre08/2 |  | 2009 | Germany: Krefeld | rat |  | KC813505 | 924 |
|  | CPXV RatGer09/1 |  | 2009 | Germany: Germering | rat |  | KC813503 | 924 |
|  | CPXV CEPAD 332 |  | 2011 | France: Epinal | human | KC592396 | This study | 924 |
|  | CPXV CEPAD 333 |  | 2011 | France: Epinal | human | KC592397 | This study | 924 |
|  | CPXV CEPAD 336 |  | 2011 | France: Epinal | human | KC592399 |  | 924 |
|  | CPXV HumLan08/1 | C030 | 2008 | Germany: Landau | human | GQ260460 | KC813492 | 921 |
|  | CPXV MonKre08/1 |  | 2008 | Germany: Krefeld | mongoose | GQ260457 |  | 921 |
|  | CPXV MonKre08/2 |  | 2008 | Germany: Krefeld | mongoose | GQ281042 |  | 921 |
|  | CPXV MonKre08/3 |  | 2008 | Germany: Krefeld | mongoose | GQ260458 |  | 921 |
|  | CPXV MonKre08/4 |  | 2008 | Germany: Krefeld | mongoose |  | KC813500 | 921 |
|  | CPXV JagKre08/1 |  | 2008 | Germany: Krefeld | jaguarundi | GQ260459 | KC813497 | 921 |
|  | CPXV JagKre08/2 |  | 2008 | Germany: Krefeld | jaguarundi |  | KC813498 | 921 |
|  | CPXV RatHei09/01 |  | 2009 | Germany: Heidelberg | rat |  | KC813504 | 921 |
|  | CPXV IT Llama 09 |  | 2009 | Italy: Viterbo | lama | HM104709 |  | 921 |
|  | CPXV-OPV 98/3 | C031 | 1998 | Germany: Braunschweig | cat | AY902270 |  | 918 |
|  | CPXV-Callithrix MKY2002 | C032 | 2002 | Germany: Lower Saxony | marmoset | AY298785 | HQ420898 | 921 |
|  | CPXV-K2984 | C033 | 2000 | United Kingdom | cat | AY902295 | HQ420900 | 894 |
|  | CPXV-Brighton Red | C034 | 1939 | United Kingdom | human |  | AF482758 | 894 |
|  | CPXV-Catpox 5 |  | 1982 | United Kingdom | cheetah | AY902254 |  | 894 |
|  | CPXV-K2739 | C035 | 2000 | United Kingdom | cat | AY902289 | KY549149 | 894 |
|  | CPXV-K428 |  | 2000 | United Kingdom | cat | AY902290 | KY549145 | 894 |
|  | CPXV-K4207 |  | 2000 | United Kingdom | cat | AY902291 | KY549150 | 894 |
|  | CPXV-Catpox 3L97 |  | 1988 | United Kingdom | cat |  | KY549143 | 894 |
|  | CPXV-K1639 | C036 | 2000 | United Kingdom | cat | AY902294 | KY549148 | 897 |
|  | CPXV-catpox3 | C037 | 1983 | United Kingdom | cat | AY902276 |  | 900 |
|  | CPXV-OPV 89/3 | C038 | 1989 | Germany: Calw | cat | AY902257 |  | 915 |
|  | CPXV-OPV 98/1 | C039 | 1998 | Germany: Landshut | human | AY902268 |  | 915 |
|  | CPXV-OPV 88 Lunge lidil | C040 | 1988 | Germany: Teissendorf | cat | AY902256 |  | 915 |
|  | CPXV-Norway human | C041 | 1994 | Norway: Bergen | human | AY902274 | HQ420899 | 915 |
|  | CPXV No-F2 |  | 1999 | Norway | cat | FJ769351 |  | 915 |
|  | CPXV No-H1 |  | 1999 | Norway | cat | FJ769352 |  | 915 |
|  | CPXV-Norway feline | C042 | 1994 | Norway | cat | AY902275 | KY549151 | 915 |
|  | CPXV-No-F1 |  | 1994 | Norway | cat | FJ769350 |  | 915 |
|  | CPXV-Bordeaux | C043 | 2010 | France: Bordeaux | human | HQ634150 |  | 915 |
|  | CPXV No-H2 | C044 | 2001 | Norway | human | FJ769353 |  | 945 |
|  | CPXV-659 | C045 | 1972x | Germany: Berlin |  | AY902299 |  | 936 |
|  | CPXV-922-99 | C046 | 1999 | Germany | cat | AF377885 |  | 939 |
|  | CPXV-EP-4 lidil |  | 1980 | Germany: Hameln | elephant | AY902280 | HQ420895 | 939 |
|  | CPXV-K779 |  | 2000 | United Kingdom | cat | AY902281 | KY549146 | 939 |
|  | CPXV-K780 |  | 2000 | United Kingdom | cat | AY902282 | KY549147 | 939 |
|  | CPXV-EP-Hannover |  | 1980 | Germany: Hannover | elephant | AY902292 |  | 939 |
|  | CPXV-75/01 |  | 2001 | Germany: Detmold | human | AY902293 |  | 939 |
|  | CPXV HumPad07/1 |  | 2007 | Germany: Paderborn | human |  | KC813496 | 939 |
|  | CPXV Katze Sammy |  | 2015 | Germany: Hannover | cat | MF948143 |  | 939 |
|  | CPXV Katze Leo |  | 2015 | Germany: Hannover | cat | MF948139 |  | 939 |
|  | CPXV Katze Cookie |  | 2015 | Germany: Hannover | cat | MF948138 |  | 939 |
|  | CPXV Katze Moritz |  | 2015 | Germany: Hannover | cat | MF948141 |  | 939 |
|  | CPXV Katze Hannover S2216/04 |  | 2004 | Germany: Hannover | cat | MF948142 |  | 939 |
|  | CPXV-277/03 | C047 | 1999 | Germany: Frankfurt | cat | AY902301 |  | **831** |
|  | CPXV-OPV 98/5 | C048 | 1998 | Germany: Muelsen | horse | AY902272 |  | 936 |
|  | CPXV-275/03 |  | 1999 | Germany: Dresden | tapir | AY902273 |  | 936 |
|  | CPXV Ger/2015/Cat3 |  | 2015 | Germany: Vogtlandkreis | cat |  | LT896733 | 936 |
|  | CPXV-65/01 | C049 | 2001 | Germany: Regensburg | human | AY902308 |  | 948 |
|  | CPXV CatBer07/1 | C050 | 2007 | Germany: Berlin | cat |  | KC813502 | 936 |
|  | CPXV HumBer07/1 |  | 2007 | Germany: Berlin | human |  | KC813509 | 936 |
|  | CPXV HumMag07/1 |  | 2007 | Germany: Magdeburg? | human |  | KC813495 | 936 |
|  | CPXV HumGri07/1 | C051 | 2007 | Germany: Grimmen | human |  | KC813511 | 921 |
|  | CPXV EleGri07/1 |  | 2007 | Germany: Grimmen | elefant |  | KC813507 | 921 |
|  | CPXV CatPot07/1 | C052 | 2007 | Germany: Potsdam | cat |  | KC813506 | 936 |
|  | CPXV HumLit08/1 | C053 | 2008 | Litauen : Vilnius | human |  | KC813493 | 948 |
|  | CPXV-HumLue09/1 | C054 | 2009 | Germany: Lübeck | human |  | KC813494 | 903 |
|  | CPXV MarLei07/1 | C055 | 2007 | Germany: Leipzig | mara |  | KC813499 | 918 |
|  | CPXV Dina | C056 | 2007 | France: Caen | human | MF948144 |  | 915 |
|  | CPXV 2012/1 | C057 | 2012 | Germany: Essen | human | KT182068 |  | 924 |
|  | CPXV Freiburg | C058 | 2014 | Germany: Freiburg | human |  |  |  |
|  | CPXV CEPAD335 | C059 | 2011 | France: Epinal | human | KC592398 |  | 918 |
|  | ITA-pox 1 | C060 | 2005 | Italy: Udine | human | EF612709 |  | 945 |
|  | CPXV Katze Maurer | C061 | 2015 | Germany: Haar | cat | MF948145 |  | 915 |
|  | CPXV Katze Mikos | C062 | 2015 | Germany: Garbsen | cat | MF948140 |  | 924 |
|  | CPXV Kostroma/2015 | C063 | 2015 | Russia | human |  | KU861503 | 939 |
|  | CPXV Amadeus 2015 | C064 | 2015 | Germany | horse |  | LN879483 | 942 |
|  | CPXV Ger/2010/Alpaca | C065 | 2010 | Germany: Oberwiesenthal | alpaca |  | LT896718 | 951 |
|  | CPXV Ger/2010/Raccoon | C066 | 2010 | Germany: Ellrich | raccoon |  | LT896730 | 960 |
|  | CPXV Ger/2007/Vole | C067 | 2007 | Germany: Rottweil (Heuberg) | vole |  | LT896722 | 948 |
|  | CPXV Ger/2013/Alpaca | C068 | 2013 | Germany: Zernitz | alpaca |  | LT896719 | 936 |
|  | CPXV Ger/2017/Alpaca2 | C069 | 2017 | Germany: Merzdorf | alpaca |  | LT896732 | 816 |
|  | CPXV Ger/2015/Cat2 | C070 | 2015 | Germany: Rostock | cat |  | LT896727 | 921 |
|  | CPXV Ger/2010/Rat | C071 | 2010 | Germany: Hannover | rat |  | LT896728 | 924 |
|  | CPXV Ger/2014/Cat1 |  | 2014 | Germany: Bleckede | cat |  | LT896723 | 924 |
|  | CPXV Ger/2015/Cat1 | C072 | 2015 | Germany: Vogtlandkreis | cat |  | LT896724 | 915 |
|  | CPXV Ger2010 MKY | C073 | 2010 | Germany: Bad Liebenstein | Cotton-top tamarin | KC493623 | LT896721 | 1032 |
|  | CPXV 14470_14 | C074 | 2014 | Germany: Giessen | human | MF948147 |  | 960 |
|  | CPXV OPV 2016/1 | C075 | 2016 | Germany: Maifeld | human | MF948146 |  | 921 |
|  | CPXV CheGrey_DK_2010 | C076 | 2010 | Denmark | cheetah |  | KY569021 | 903 |
|  | CPXV CheTopCut_DK_2011 | C077 | 2011 | Denmark | cheetah |  | KY569022 | 903 |
|  | CPXV CheNuru_DK_2012 |  | 2012 | Denmark | cheetah |  | KY569020 |  |
|  | CPXV CheNova_DK_2014 |  | 2014 | Denmark | cheetah |  | KY569019 |  |
|  | CPXV CheHurley_DK_2012 |  | 2012 | Denmark | cheetah |  | KY569018 |  |
|  | CPXV Ger/2015_human Bauer | C78 | 2015 | Germany | human |  |  | 942 |
|  | CPXV-Katzenbaer(HA14) | C79 | 1997 | Germany: Berlin | lesser panda | AY902261 |  | 936 |
|  | CPXV-BeaBer04/1 |  | 2004 | Germany: Berlin | beaver |  | KC813491 | 936 |
|  | CPXV-EP-6 cow(HA34) |  | 1998 | Germany: Berlin | elephant | AY902262 |  | 936 |
| Taterapox | TATV-gbl-pox | T001 | 1968 | Benin | gerbil | AF375093 | DQ437594 | 960 |
| Camelpox | CMLV-pox | L001 | 1978 | Somalia | camel | AF375081 |  | 948 |
|  | CMLV-Syria |  | 2005 | Syrien | camel | DQ853384 |  | 948 |
|  | CMLV-Yabello (n=10) |  | 2014 | Ethiopia | camel | KU645590 |  | 948 |
|  | CMLV-Miyu (n=8) |  | 2014 | Ethiopia | camel | KU645580 |  | 948 |
|  | CMLV-Hordha (n=2) |  | 2011 | Ethiopia | camel | KU645572 |  | 948 |
|  | CMLV-Hadow (n=2) |  | 2012 | Ethiopia | camel | KU645570 |  | 948 |
|  | CMLV-Golajo (n=3) |  | 2012 | Ethiopia | camel | KU645568 |  | 948 |
|  | CMLV-Fafan (n=2) |  | 2012 | Ethiopia | camel | KU645565 |  | 948 |
|  | CMLV-M-96 Kazakhstan | L002 | 1996 | Kazakhstan | camel |  | AF438165 | 960 |
|  | CMLV_CMS | L003 | 1970 | Iran | camel |  | AY009089 | 948 |
|  | CMLV-saudi |  | 1986 | Saudi Arabia | camel | AF375082 |  | 948 |
|  | CMLV-cp-5 |  | 1992 | United Arab Emirates | camel | AF375080 |  | 948 |
|  | CMLV-CP-18 |  | 1994 | United Arab Emirates | camel | AY902247 |  | 948 |
|  | CMLV-CP-28 |  | 1994 | United Arab Emirates | camel | AY902248 |  | 948 |
|  | CMLV-CP 266/96 |  | 1996 | United Arab Emirates | camel | AY902249 |  | 948 |
|  | CMLV-CP-298-2 |  |  | United Arab Emirates | camel | AY902250 |  | 948 |
|  | CMLV-202-95 Haut |  | 1995 | Saudi Arabia | camel | AY902251 |  | 948 |
|  | CMLV0408151v |  | 2015 |  | camel |  | KP768318 | 948 |
| Variola major | VARV-IND3_1967 | S001 | 1967 | India | human |  | [NC_001611](http://www.ncbi.nlm.nih.gov/entrez/viewer.fcgi?db=nuccore&id=NC_001611) | 942 |
|  | VARV-TZA65 | S002 | 1965 | Tanzania | human | AY944044 | [DQ441443](http://www.ncbi.nlm.nih.gov/entrez/viewer.fcgi?db=nuccore&id=DQ441443) | 942 |
|  | VARV-ilm |  | 1965 | Tanzania | human | AF375135 |  | 942 |
|  | VARV-ETH72_16 |  | 1972 | Ethiopia | human | AF375132 | [DQ441424](http://www.ncbi.nlm.nih.gov/entrez/viewer.fcgi?db=nuccore&id=DQ441424) | 942 |
|  | VARV-ETH72_17 |  | 1972 | Ethiopia | human | AF375133 | [DQ441425](http://www.ncbi.nlm.nih.gov/entrez/viewer.fcgi?db=nuccore&id=DQ441425) | 942 |
|  | VARV-COG70_46 |  | 1970 | Democratic Republic of Congo | human |  | [DQ437583](http://www.ncbi.nlm.nih.gov/entrez/viewer.fcgi?db=nuccore&id=DQ437583) | 942 |
|  | VARV-COG70_227 |  | 1970 | Democratic Republic of Congo | human |  | [DQ441423](http://www.ncbi.nlm.nih.gov/entrez/viewer.fcgi?db=nuccore&id=DQ441423) | 942 |
|  | VARV-BWA72 |  | 1972 | Botswana | human |  | [DQ441417](http://www.ncbi.nlm.nih.gov/entrez/viewer.fcgi?db=nuccore&id=DQ441417) | 942 |
|  | VARV-BWA73 |  | 1973 | Botswana | human |  | [DQ441418](http://www.ncbi.nlm.nih.gov/entrez/viewer.fcgi?db=nuccore&id=DQ441418) | 942 |
|  | VARV-ZAF65_102 |  | 1965 | Republic of South Africa | human | AY944038 | [DQ44143](http://www.ncbi.nlm.nih.gov/entrez/viewer.fcgi?db=nuccore&id=DQ441436)5 | 942 |
|  | VARV-ZAF65_103 |  | 1965 | Republic of South Africa | human |  | [DQ441436](http://www.ncbi.nlm.nih.gov/entrez/viewer.fcgi?db=nuccore&id=DQ441436) | 942 |
|  | VARV-DEU58 |  | 1958 | Germany: Heidelberg | human | AY944039 | [DQ437584](http://www.ncbi.nlm.nih.gov/entrez/viewer.fcgi?db=nuccore&id=DQ437584) | 942 |
|  | VARV-GBR47_hig |  | 1947 | United Kingdom | human | AY944040 | [DQ441446](http://www.ncbi.nlm.nih.gov/entrez/viewer.fcgi?db=nuccore&id=DQ441446) | 942 |
|  | VARV-GBR46_hind |  | 1946 | United Kingdom | human | AY944041 | DQ441445 | 942 |
|  | VARV-tlv |  | 1958 | United Kingdom | human | AF375144 |  | 942 |
|  | VARV-CHN48 |  | 1948 | China | human | AY944042 | [DQ437582](http://www.ncbi.nlm.nih.gov/entrez/viewer.fcgi?db=nuccore&id=DQ437582) | 942 |
|  | VARV-IND53_mad |  | 1953 | India | human | AY944043 | [DQ441427](http://www.ncbi.nlm.nih.gov/entrez/viewer.fcgi?db=nuccore&id=DQ441427) | 942 |
|  | VARV-KOR47 |  | 1947 | Korea | human | AY944045 | [DQ441432](http://www.ncbi.nlm.nih.gov/entrez/viewer.fcgi?db=nuccore&id=DQ441432) | 942 |
|  | VARV-SDN47_rum |  | 1947 | Sudan | human |  | DQ441441 | 942 |
|  | VARV-SDN47_jub |  | 1947 | Sudan | human | AY944046 | DQ441440 | 942 |
|  | VARV-SUM70_222 |  | 1970 | Sumatra | human | AY944047 | [DQ437591](http://www.ncbi.nlm.nih.gov/entrez/viewer.fcgi?db=nuccore&id=DQ437591) | 942 |
|  | VARV-SUM70_228 |  | 1970 | Sumatra | human |  | [DQ4](http://www.ncbi.nlm.nih.gov/entrez/viewer.fcgi?db=nuccore&id=DQ437591)41442 | 942 |
|  | VARV-India 71 |  | 1971 | India | human | EF611229 |  | 942 |
|  | VARV-Bombay |  | 1958 | India | human | AF375128 |  | 942 |
|  | VARV-Helder |  | 1962 | Tanzania | human | EF611226 |  | 942 |
|  | VARV-13/62 |  | 1962 | Tanzania | human | EF611221 |  | 942 |
|  | VARV-Ngami |  | 1962 | Tanzania | human | EF611217 |  | 942 |
|  | VARV-Wzim Ahmed |  | 1970 | Pakistan | human | EF611219 |  | 942 |
|  | VARV-Aziz |  | 1970 | Pakistan | human | EF611222 |  | 942 |
|  | VARV-af2 |  | 1972 | Afghanistan | human | AF375126 |  | 942 |
|  | VARV-af3 |  | 1971 | Afghanistan | human | AF375127 |  | 942 |
|  | VARV-Rw-18 |  | 1970 | Rwanda | human | EF611218 |  | 942 |
|  | VARV-Mary |  | 1962 | Tanzania | human | EF611212 |  | 942 |
|  | VARV-cng |  | 1970 | Kongo | human | AF375131 |  | 942 |
|  | VARV V563 |  |  | CZ | human |  | LT706528 | 942 |
|  | VARV-SOM77_1252 | S003 | 1977 | Somalia | human |  | [DQ441438](http://www.ncbi.nlm.nih.gov/entrez/viewer.fcgi?db=nuccore&id=DQ441438) | 942 |
|  | VARV-SOM77_1605 |  | 1977 | Somalia | human |  | [DQ441439](http://www.ncbi.nlm.nih.gov/entrez/viewer.fcgi?db=nuccore&id=DQ441439) | 942 |
|  | VARV-SOM77_ali |  | 1977 | Somalia | human |  | DQ437590 | 942 |
|  | VARV-SOM |  | 1977 | Somalia | human | AF375143 |  | 942 |
|  | VARV-GBR44_harv | S004 | 1946 | United Kingdom | human |  | DQ441444 | 942 |
|  | VARV-PAK_1969 | S005 | 1969 | Pakistan | human | AY944052 | [DQ437589](http://www.ncbi.nlm.nih.gov/entrez/viewer.fcgi?db=nuccore&id=DQ437589) | 942 |
|  | VARV-AFG70 |  | 1970 | Afghanistan | human | AY944048 | [DQ437580](http://www.ncbi.nlm.nih.gov/entrez/viewer.fcgi?db=nuccore&id=DQ437580) | 942 |
|  | VARV-IND64_vel4 |  | 1964 | India | human | AY944049 | [DQ437585](http://www.ncbi.nlm.nih.gov/entrez/viewer.fcgi?db=nuccore&id=DQ437585) | 942 |
|  | VARV-IRN72 |  | 1972 | Iran | human | AY944050 | [DQ437587](http://www.ncbi.nlm.nih.gov/entrez/viewer.fcgi?db=nuccore&id=DQ437587) | 942 |
|  | VARV-KWT67 |  | 1966 | Kuwait | human | AY944051 | [DQ441433](http://www.ncbi.nlm.nih.gov/entrez/viewer.fcgi?db=nuccore&id=DQ441433) | 942 |
|  | VARV-SYR72 |  | 1972 | Syria | human | AY944053 | [DQ437592](http://www.ncbi.nlm.nih.gov/entrez/viewer.fcgi?db=nuccore&id=DQ437592) | 942 |
|  | VARV-YUG72 |  | 1972 | Yugoslavia | human | AY944054 | [DQ441448](http://www.ncbi.nlm.nih.gov/entrez/viewer.fcgi?db=nuccore&id=DQ441448) | 942 |
|  | VARV-Kuw-5 |  | 1967 | Kuwait | human | [EF611209](http://www.ncbi.nlm.nih.gov/entrez/viewer.fcgi?db=nuccore&id=DQ441448) |  | 942 |
|  | VARV-rat |  | 1969 | Pakistan | human | [AF375141](http://www.ncbi.nlm.nih.gov/entrez/viewer.fcgi?db=nuccore&id=DQ441448) |  | 942 |
|  | VARV-Madras |  | 1962 | Indien | human | [AF3751](http://www.ncbi.nlm.nih.gov/entrez/viewer.fcgi?db=nuccore&id=DQ441448)37 |  | 942 |
|  | VARV-BGD75maj | S006 | 1975 | Bangladesh | human |  | L22579 | 942 |
|  | VARV-BGD74_nur |  | 1974 | Bangladesh | human | AF375139 | [DQ441420](http://www.ncbi.nlm.nih.gov/entrez/viewer.fcgi?db=nuccore&id=DQ441420) | 942 |
|  | VARV-BGD74_shz |  | 1974 | Bangladesh | human |  | [DQ44142](http://www.ncbi.nlm.nih.gov/entrez/viewer.fcgi?db=nuccore&id=DQ441420)1 | 942 |
|  | VARV-BGD74_sol |  | 1974 | Bangladesh | human |  | [DQ44142](http://www.ncbi.nlm.nih.gov/entrez/viewer.fcgi?db=nuccore&id=DQ441420)2 | 942 |
|  | VARV-BGD75_Banu |  | 1975 | Bangladesh | human |  | DQ437581 | 942 |
|  | VARV-aba |  | 1974 | Bangladesh | human | AF375125 |  | 942 |
|  | VARV-hawa |  | 1974 | Bangladesh | human | AF375134 |  | 942 |
|  | VARV-jal |  | 1974 | Bangladesh | human | AF375136 |  | 942 |
|  | VARV-par |  | 1974 | Bangladesh | human | AF375140 |  | 942 |
|  | VARV-NPL73 |  | 1973 | Nepal | human | AY944033 | DQ437588 | 942 |
|  | VARV-Ind-3a |  | 1967 | India | human | EF611227 |  | 942 |
|  | VARV-Ind-4a |  | 1967 | India | human | EF611228 |  | 942 |
|  | VARV-Khateen |  | 1970 | Pakistan | human | EF611230 |  | 942 |
|  | VARV-JPN46_yam | S007 | 1946 | Japan | human | AY944034 | [DQ441429](http://www.ncbi.nlm.nih.gov/entrez/viewer.fcgi?db=nuccore&id=DQ441429) | 942 |
|  | VARV-JPN51_hrpr |  | 1951 | Japan | human | AY944034 | [DQ441430](http://www.ncbi.nlm.nih.gov/entrez/viewer.fcgi?db=nuccore&id=DQ441430) | 942 |
|  | VARV-IND53_ndel |  | 1953 | India | human | AY944035 | [DQ441428](http://www.ncbi.nlm.nih.gov/entrez/viewer.fcgi?db=nuccore&id=DQ441428) | 942 |
|  | VARV-JPN51_stwl |  | 1951 | Japan | human | AY944036 | [DQ441431](http://www.ncbi.nlm.nih.gov/entrez/viewer.fcgi?db=nuccore&id=DQ441431) | 942 |
|  | VARV-IND64_vel5 | S008 | 1964 | India | human | AY944037 | [DQ437586](http://www.ncbi.nlm.nih.gov/entrez/viewer.fcgi?db=nuccore&id=DQ437586) | 942 |
|  | VARV-6-58 | S009 | 1958 | Pakistan | human | EF611220 |  | 942 |
|  | VARV-M-A-60 | S010 | 1960 | Russia | human | EF611210 |  | 966 |
|  | VARV-M-Abr-60 |  | 1960 | Russia | human | EF611211 |  | 942 |
|  | VARV-M-Bl-60 |  | 1960 | Russia | human | EF611213 |  | 942 |
|  | VARV-M-Gavr-60 |  | 1960 | Russia | human | EF611214 |  | 942 |
|  | VARV-M-N-60 |  | 1960 | Russia | human | EF611215 |  | 942 |
|  | VARV-M-Sur-60 |  | 1960 | Russia | human | EF611216 |  | 942 |
|  | VARV V1588 | S011 |  | CZ | human |  | LT706529 | 981 |
|  | VARV VD21 | S012 |  | Lithania | human (mummy) |  | KY358055 | 957 |
| Variola minor | VARV-GBR52 but | S013 | 1952 | United Kingdom | human | AF375129 AY944057 | DQ441447 | 957 |
|  | VARV-Brazil_131 |  |  | Brazil | human | EF611223 |  | 957 |
|  | VARV-BRA66 | S014 | 1966 | Brazil | human | AY944058 | DQ441419 | 957 |
|  | VARV-cm6 |  | 1968 | Brazil | human | AF375130 |  | 957 |
|  | VARV-Gar 1966 | S015 | 1966 | Brazil | human | Y16780 |  | 957 |
|  | VARV-NER69 | S016 | 1969 | Niger | human |  | DQ441434 | 957 |
|  | VARV-BEN68 |  | 1968 | Benin | human | AY944055 | DQ441416 | 957 |
|  | VARV-nig |  | 1961 | Nigeria | human | AF375138 |  | 957 |
|  | VARV-SLE68 | S017 | 1968 | Sierra Leone | human | [AF375142](http://www.ncbi.nlm.nih.gov/entrez/viewer.fcgi?db=nuccore&id=DQ441426) | [DQ441437](http://www.ncbi.nlm.nih.gov/entrez/viewer.fcgi?db=nuccore&id=DQ441437) | 957 |
|  | VARV-GIN69 |  | 1969 | Guinea | human | AY944056 | [DQ441426](http://www.ncbi.nlm.nih.gov/entrez/viewer.fcgi?db=nuccore&id=DQ441426) | 957 |
| Raccoonpox | RCNV-85a | R001 | 1964 | USA: Maryland | raccoon | AF375116 |  | 933 |
|  | RCNV-84 |  | 1964 | USA: Maryland | raccoon | AF375115 |  | 933 |
|  | RCNV-85b |  | 1964 | USA: Maryland | raccoon | AY375117 |  | 933 |
| Skunkpox | SKKP-pox | K001 | 1978 | USA: Washington | striped skunk | AF375120 |  | 942 |
| Volepox | VPXV-pox | O001 | 1987 | USA: California | California vole | AF375145 |  | 861 |
