# Supplementary material for: Use of Next Generation Sequencing to study two cowpox virus outbreaks
Source: PeerJ. 2019 Mar 1;7:e6561. doi: 10.7717/peerj.6561 (PMC6398431; doi:10.7717/peerj.6561)

Supplemental Data:

Maximum-Parsimony Dendrogram of aligned and identified HA-gene-genotypes of orthopoxviruses. Label of genotypes correspond to Suppl_Data 1. Branch lengths show nucleotide differences between the investigated strains.

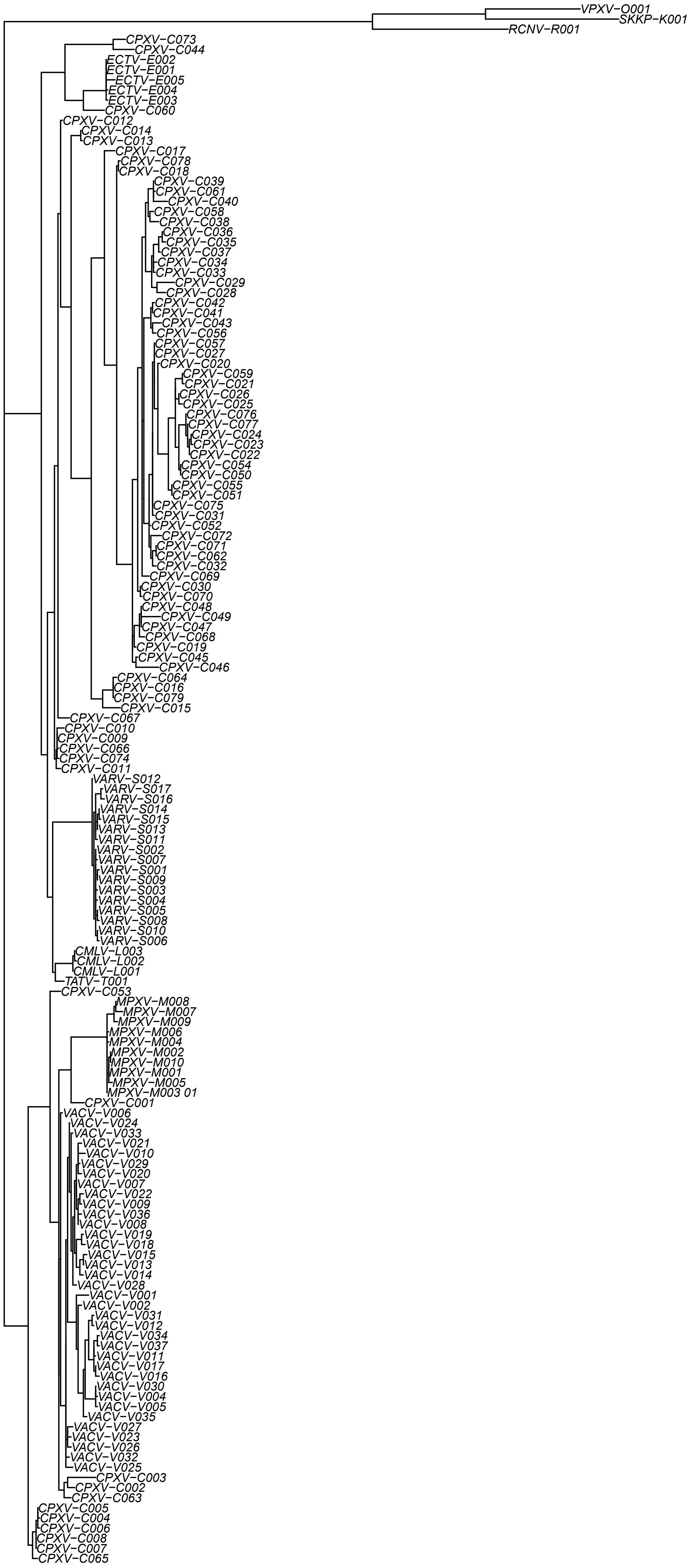

Supplement: Figure S1 — Label of genotypes correspond to Suppl_Data 1. Branch lengths show nucleotide diferences between the investigated strains. [file peerj-07-6561-s002.pdf]
